# Soil fungal community comparison of different mulberry genotypes and the relationship with mulberry fruit sclerotiniosis

**DOI:** 10.1038/srep28365

**Published:** 2016-06-21

**Authors:** Cui Yu, Xingming Hu, Wen Deng, Yong Li, Guangming Han, Chuhua Ye

**Affiliations:** 1 Industrial Crops Institute of Hubei Academy of Agricultural Sciences, Wuhan 430064, China

## Abstract

Mulberry fruit sclerotiniosis is a common soil-borne disease. We hypothesize that there is a relationship between the incidence of mulberry fruit sclerotiniosis and the soil fungal community. Therefore four mulberry genotypes with different resistance to sclerotiniosis were selected to study their soil fungal community under the same condition. A total of 6 phyla and 93 genera were identified from all mulberry samples. Genera affected by genotype, cover, and genotype by cover interactions, were 23, 20, and 11, respectively. There were 10 genera that differed between the resistant cultivars and the susceptible cultivars. In addition, the relative abundances of *Humicola* and *Mortierella* in the resistant mulberry cultivars with the cover treatments were significantly higher than that of in the susceptible cultivars. However, the relative abundance of *Sclerotiniaceae* and *Scleromitrula* that may cause sclerotiniosis in the uncover treatments were significantly higher compared with the cover treatments. The results suggested that the genotype of mulberry has a significant impact on the soil microbial community that may result in differences in resistance to sclerotiniosis, and covering could reduce the relative abundance of *Sclerotiniaceae* or *Scleromitrula*.

The health status of a plant is a result of complex interactions between the plant, the soil environment, and microorganisms, including pathogens and other microorganisms in the soil. Microbial diversity in the soil is one of the main components that determines soil health^1^,^2^ and is believed to be one of the main drivers in soil suppressiveness. Studies on the soil microbial communities of some crops have reported that microbial communities differed between different disease-resistant cultivars^3^,^4^,^5^,^6^. Diab El Arab *et al*.^5^ reported that the relative abundances of cy19:0 and 18:1 PLFAs, which are indicators of Gram-negative Proteobacteria, were found to be significantly different in the rhizosphere of different wheat cultivars^5^. Yao and Wu^6^ found that the relative abundances of 18:2ω6,9c, 16:0 and 15:0a PLFAs were also altered by different cucumber cultivars^6^. In addition, more soil pathogenic microorganisms were found in the soil of the susceptible soybean cultivar than in the soil of the resistant cultivar^7^. Most studies on the role of plant genotypes on soil-borne disease analyse only a specific group of pathogenic microorganisms. However, minimal information is available on the investigation of how plants regulate the whole microbial community in the soil.

Mulberry fruits are highly nutritious, have a unique flavour, and have many medicinal properties. In recent years, due to the popularly of mulberry fruit for sightseeing and picking and series of health products in the market, the economic value of the mulberry fruit has risen. The planting area of the mulberry is more than 20 thousand hectares in the Hubei province and has been developed and expanded year after year, but, as a consequence, the occurrence of mulberry fruit sclerotiniosis has become more and more serious. The lighter results of this disease can reduce the output to 30–50%, and the severe results cut output by 80% or even lead to no harvest, seriously affecting the economic benefits of planting mulberry. Previous studies have reported that sclerotiniosis is caused by *Scleromitrula* or *Sclerotinia* which is distributed worldwide and causes disease in mulberry and many other important crops^8^,^9^,^10^,^11^,^12^,^13^. However, the impact of mulberry genotype on the soil fungal community and the relationship with sclerotiniosis was not studied so far. So four mulberry (*Morus* SPP.) genotypes Da 10 (DS), Yunguo 1 (YG), Dabaie (DB), and Taiwan 72C002 (TW) treated with cover (C) and no-cover (NC) with different resistance to sclerotiniosis were selected for this study. When sampling, mulberry fruit sclerotiniosis was occurring in all tested plants. However, the incidence of sclerotiniosis was different. DS is susceptible to the mulberry fruit sclerotiniosis, while YG, DB and TW are resistant to the mulberry fruit sclerotiniosis (Table 1). Moreover, in the production, the prevention and control of sclerotiniosis mainly are chemical fungicides. Uncontrolled use of chemical fungicides has severely affected the agroecosystem, associated with environmental and health hazards. So using the comprehensive measures to prevention and control of sclerotiniosis is very important. Covering has been suggested as an important agronomic measure to prevent the sclerotiniosis of mulberry fruits. But there was not any research studying the relationship between covering and soil fungal community, especially for the pathogens.

The aim of this study was to characterize the soil fungal communities to answer: Does the overall soil fungal community differ between the soils in the resistant cultivar and in the susceptible cultivar? Can cover treatments affect the soil fungal community, especially for the pathogens? This information will improve our understanding of the relationship between the soil microbial ecology and soil-borne disease.

## Results

### DNA Sequence data and fungal community

A total of 923,697 of ITS1 sequences reads from the 24 samples with an average of 38,487 sequences reads for each sample were used for this project. After initial quality control, 747,854 high quality sequences were obtained. Based on 97% species similarity, 384–684 operational taxonomic units (OTUs) were separately obtained from samples of different mulberry genotypes (see Supplementary Table S1 online). The average length of the sequence reads was 254 bp, and they were classified into different taxonomies using uclust^14^. The taxon abundance of each sample was distributed into phylum, class, order, family, genus, and species levels using Unite (Release 5.0, http://unite.ut.ee/index.php)^15^.

**Figure 1:**
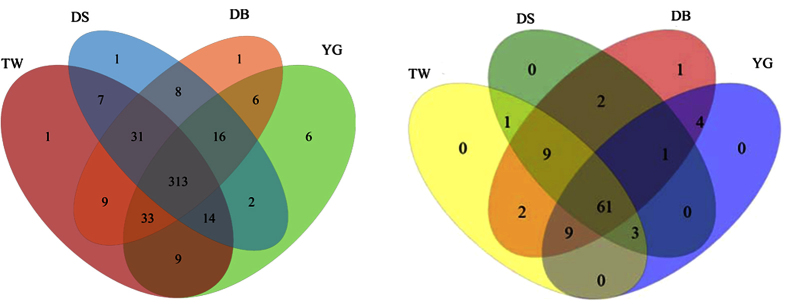
A Venn diagram was generated to describe the common and unique OTUs (**a**) and Genera (**b**) among the susceptible cultivar Da 10 (DS), resistant cultivar Yunguo 1 (YG), Taiwan 72C002 (TW), and Dabaie (DB).

Both the abundance and diversity of fungi in mulberry field soil were correlated with the genotype. From the Chao1 index and Shannon index, we found that fungal community diversity in the sclerotiniosis-susceptible DS soil were significantly lower than that of in other mulberry soils (p < 0.05; Supplementary Fig. S1). A Venn diagram of the four genotypes indicated that the number of OTUs in the sclerotiniosis-susceptible DS and sclerotiniosis-resistant YG, TW, DB groups with cover treatment was 365, 399, 417 and 417, respectively. A total of 313 OTUs were shared by the four different genotypes (Fig. 1a). The principal component analysis (PCA) of soil fungal genera showed that the soil samples were separated into categories, which matched their mulberry genotypes (Supplementary Fig. S2a). The separation was clearer for the susceptible cultivar of DS than the resistant cultivars of YG, DB and TW, suggesting that the genotype influenced the soil fungi community.

**Figure 2:**
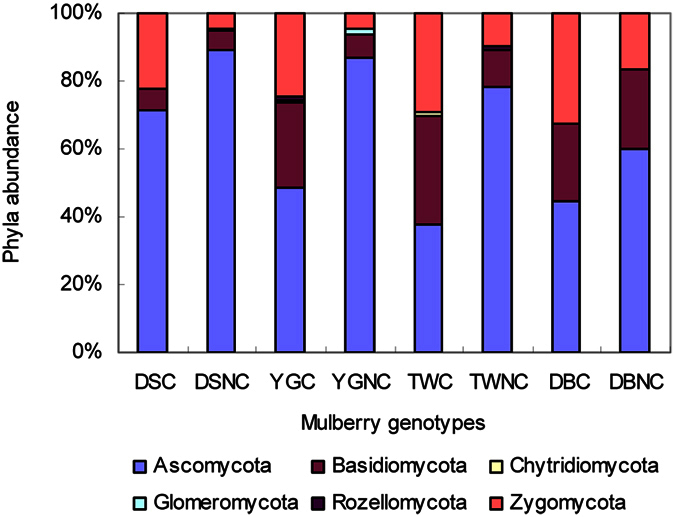
Phylum distribution of the susceptible cultivar Da 10 (DS), resistant cultivar Yunguo 1 (YG), Taiwan 72C002 (TW), and Dabaie (DB) with the covered (C) and no-covered (NC) treatments.

The results shown in Fig. 2 describe the distribution of the DNA sequences into phyla. A total of six phyla were shared by the all soil samples, as follows: *Ascomycota*, *Basidiomycota*, *Chytridiomycota*, *Glomeromycota*, *Rozellomycota* and *Zygomycota*. *Ascomycota* was the most dominant, regardless of the different samples, and comprised more than 63% of the total sequences. Two phyla (*Ascomycota* and *Basidiomycota*) differed (p < 0.05) between the resistant mulberry cultivar and the susceptible mulberry cultivar (Table 2). *Ascomycota* in the sclerotiniosis-susceptible DS soils were higher than in other mulberry soils. In addition, *Ascomycota* was more abundant in the uncovered soil than in the covered soil among the different mulberry cultivars. In the covered soils of the sclerotiniosis-resistant genotypes (YGC, TWC and DBC), a higher percentage (25.3%, 32.1% and 23.4%, respectively) of the sequences was assigned to *Basidiomycota.*

**Figure 3:**
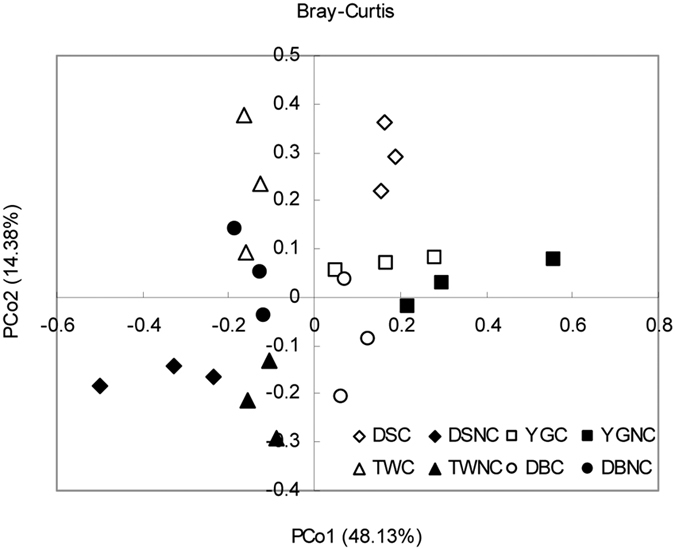


At the genus level, a total of 93 genera were identified from all samples, regardless of genotype and cover treatment. The number of genera in the DS, YG, TW and DB soils with cover treatment was 77, 78, 85 and 89, respectively. A total of 61 genera were shared by the four different genotypes (Fig. 1b). Of these 93 genera, 31 were affected by genotype, cover, and genotype by cover interactions (p < 0.05). Among these 31 genera, 23 genera were affected by genotype, 20 by cover, and 11 by the genotype by cover interaction (Table 3, Supplementary table S2). There were 10 genera that differed for the resistant cultivar and the susceptible cultivar (Table 4, Supplementary Table S3), 6 belong to *Ascomycota*. 6, 7 and 11 genera differed between the sclerotiniosis-resistant DB, TW, YG and the sclerotiniosis-susceptible DS, respectively (Supplementary Table S4). The principal coordinates analysis (PCoA) of Bray-Curtis distance matrices indicated that the variation was explained by the genotype and cover treatment (Fig. 3). The first two principal coordinates, which explains more than 60% of the variation in the data, clearly separates different genotypes and cover treatments (especially for DSC and DSNC soils), suggests that the genotype and cover treatment influenced the community of the soil fungi.

**Figure 4:**
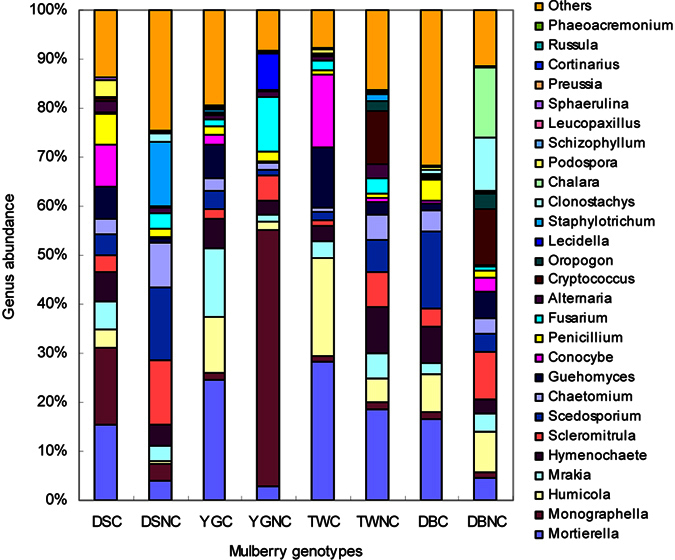
Genus distribution of the susceptible cultivar Da 10 (DS), resistant cultivar Yunguo 1 (YG), Taiwan 72C002 (TW), and Dabaie (DB) with the covered (C) and no-covered (NC) treatments.

**Figure 5:**
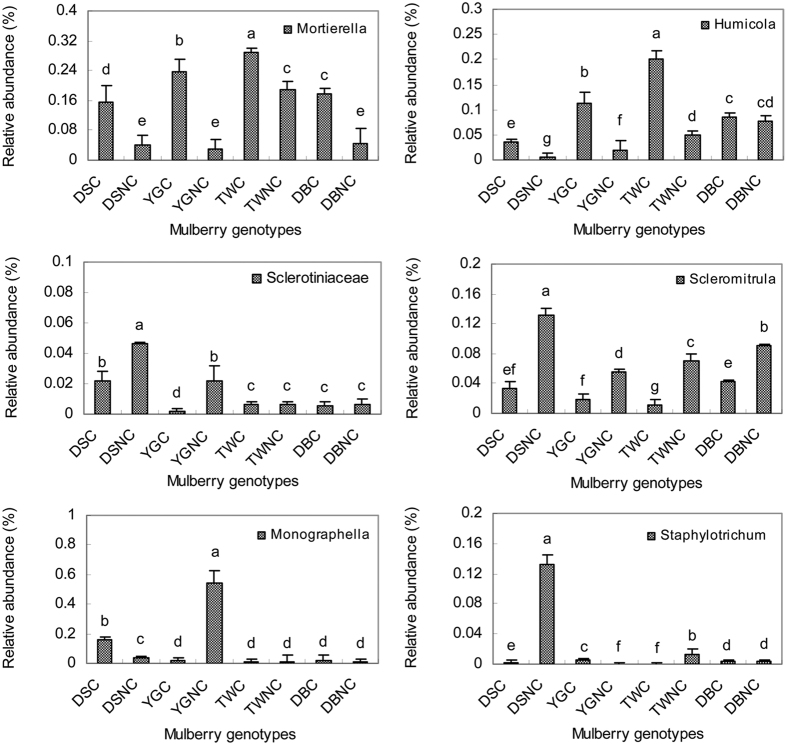
The relative abundances of *Mortierella* (a), *Humicola* (b), *Sclerotiniaceae* (c), *Scleromitrula* (d), *Monographella* (e) and *Staphylotrichum* (f) in the susceptible cultivar Da 10 (DS), resistant cultivar Yunguo 1 (YG), Taiwan 72C002 (TW), and Dabaie (DB) with the covered (C) and no-covered (NC) treatments. The error bars indicate the standard deviation (SD) (n = 3). Different letters above the bars denote a statistical significance at p < 0.05, according to LED’s tests.

The seven most abundance genera (containing more than 64% of the total sequences), namely, *Mortierella*, *Monographella*, *Humicola*, *Scedosporium*, *Scleromitrula*, *Hymenochaete* and *Mrakia*, were significantly affected by genotype and cover treatment (Figs 4 and 5, Supplementary Fig. S3). The dominant genus was *Mortierella* (17.3%) followed by *Monographella* (11.9%), *Humicola* (8.8%), *Scedosporium* (7.9%), *Scleromitrula* (6.8%), *Hymenochaete* (6.3%) and *Mrakia* (5.7%). The relative abundances of *Mortierella* and *Humicola* in the soil of resistant mulberry cultivars (YGC, TWC and DBC) were significantly higher than that of in the soil of the susceptible cultivar (DSC) (p < 0.05, Fig. 5a,b), and those in the covered treatments were significantly higher than those in the uncovered treatments (p < 0.05, except *Humicola* in DB soil). However, the relative abundances of *Sclerotiniaceae* and *Scleromitrula* in the soil of susceptible mulberry cultivar (DSNC) were significantly higher compared with the soils of the resistant mulberry cultivars (p < 0.05, Fig. 5c,d), indicating pathogen accumulation. Moreover, the abundance of *Staphylotrichum* in DSNC soil was 28.6 to 214.8-fold higher compared with other mulberry soils (p < 0.05, Fig. 5f).

### Cover treatment has a significant effect on the fungal community

Regardless of mulberry genotypes, 18 genera were differed between cover and uncover treatments, and 13 belongs to the phylum *Ascomycota* (p < 0.05, Table 5). However, in the sclerotiniosis-susceptible DS mulberry, 22 genera were different (p < 0.05) between the covered and uncovered treatments, as were 11 genera in the DB mulberry (see Supplementary Table S5). Our data suggested that there were more different genera in the soil of the susceptible mulberry between the covered and uncovered treatments. Among the different genera, the relative abundances of *Sclerotiniaceae* and *Scleromitrula* in the covered treatments were significantly lower than that of in the uncovered treatments, which suggested that the covered treatment changes the abundance of *Sclerotiniaceae* and *Scleromitrula*, which are the main genera affecting sclerotiniosis. The soil samples could be separated into categories, which matched the covered and uncovered treatments (Supplementary Fig. S2b), suggesting that covering influences the community of the soil fungi.

## Discussion

The main goal of this study was to preliminarily reveal the changes in soil fungal community of different mulberry genotypes and the relationship with mulberry fruit sclerotiniosis. Because external and inner factors can influence this complex soil ecosystem^16^,^17^, they could dilute or mask the impact of the mulberry’s genotypes. In addition, because of the uncertainties in sample collection and microbial analyses, our experiment included only four mulberry cultivars grafted onto a common rootstock prior to planting and grown in the same soil, and this is a limitation to confirm the effect of plant genotype on soil microbial community. However, the present study revealed that mulberry genotypes had a statistically significant impact on soil fungal community composition and abundance. When analysing the OTUs at the phylum level, in all of the soil samplings, *Ascomycetes* were dominant, followed by *Basidiomycetes*. This result is in accordance with the findings of previous studies^18^,^19^,^20^ that also investigated soil fungal communities using deep amplicon sequencing. However, these two phyla (*Ascomycota* and *Basidiomycota*) differed (p < 0.05) between the resistant mulberry cultivar and the susceptible mulberry cultivar (Table 2). We also found that the abundance of *Ascomycetes* in the soils of the susceptible cultivar was higher than that of in the soils of the resistant cultivar and that the abundance in the uncovered soils was higher than that of in the covered soils. These results suggested that the soil fungal abundance of the resistant cultivar and the susceptible cultivar at the phyla level were different and that the cover treatment influences the soil fungal abundance.

At the genus level, the Chao 1 index, the Shannon index and the numbers of OTUs in the soils of the resistant cultivars were higher than those of in the susceptible cultivar soils (Fig. 1a, Supplementary Fig. S1). Of the 93 fungi genera, 23 were significantly affected by mulberry genotypes, 20 by cover, and 11 by the genotype by cover interaction (Table 3, Supplementary Table S2). 10 genera differed between the resistant cultivar and the susceptible cultivar (Table 4, Supplementary Table S3). In addition, the abundances of dominant genera *Mortierella* and *Humicola* in the susceptible mulberry soils were significantly lower than that of in the resistant mulberry soils, while the relative abundances of *Staphylotrichum* was significantly increased in susceptible mulberry soils (Fig. 5). The above results suggested that genotypes did affect the soil fungal community diversity and abundance which may affect the resistance to fungal soil-borne disease^21^. To the best of our knowledge, none of these fungi had previously been reported to be pathogenic to mulberry. In fact, *Humicola* could synthesize soil organic matter and might have induced local and systemic resistance against pathogens^22^. Therefore, it may be that the decline of beneficial fungi or fungal diversity in the susceptible mulberry cultivars reduced the biotic suppression of soil-borne diseases.

Through investigation, the mulberry genotype of DS is very susceptible to sclerotiniosis, and the mulberry genotypes of YG, TW and DB are resistance to sclerotiniosis (Table 1). Previous studies have reported that the fungi of *Scleromitrula* or *Sclerotinia* could cause sclerotiniosis^18^. Despite all tested plants experiencing sclerotiniosis at the time of sampling, fungi belonging to the *Scleromitrula* and *Sclerotinia* genera were specifically enriched in the soil of the susceptible DS cultivar. In addition, the relative abundances of these fungi significantly increased in the uncover treatment soils. Therefore, all results of our study suggest that mulberry genotype had a significant impact on the soil microbial community composition and abundance, and the differences in the soil microbial community may result in the differences in the resistance to mulberry fruit sclerotiniosis. The increase in the abundance of *Sclerotiniaceae* or *Scleromitrula* may have relationship with the incidence of mulberry fruit sclerotiniosis, but covering could reduce the abundance of *Sclerotiniaceae* or *Scleromitrula*.

## Methods

### Site description and experimental design

The experimental site was established at the experimental farm of the Industrial Crops Institute at the Hubei Academy of Agricultural Sciences in Hubei Province, China (30°35’N, 114°37’E, 50 m a.s.l.). This region has a typical subtropical monsoon climate with an average annual precipitation of 1,269 mm and an average temperature between 15.8 °C and 17.5 °C. To assess the effects of the different mulberry genotypes coupled with the covered and uncovered treatments on soil microbial properties, a completely randomized block design with three replicates was designed. Four mulberry (*Morus* SPP.) cultivars Da 10 (DS), Yunguo 1 (YG), Dabaie (DB), and Taiwan 72C002 (TW) were obtained from Hangzhou, China. DS, TG and DB are the most widely cultivated in China with good economic characters. TW is a high-yield cultivar and is widely cultivated in the Taiwan region. Four one-year-old grafted mulberry cultivars with the same rootstock were planted in 2009 with a row × line spacing of 1.0 m × 1.7 m. The area of each plot was 66.7 m^2^. Half of each plot was covered with black mulch, and the other half had no mulch. The investigated soil was classified as yellow-brown according to the China Classification System. Some of the initial characteristics of the surface (0–20 cm) soil were as follows: pH, 6.49 ± 0.05; soil organic matter (SOM), 2.21 ± 0.08%; available nitrogen, 41.0 ± 1.01 mg kg^–1^; available phosphorus, 44.2 ± 2.01 mg kg^–1^; and available potassium, 132.3 ± 3.21 mg kg^–1^.

After the mulberry was planted, organic and inorganic compound fertilizer was applied at an application rate of 3000 kg·ha^–1^. The nutrient ratio of organic-inorganic compound fertilizer was 15:4:6 for N:P_2_O_5_:K_2_O, and the organic matter content was 20%. The fertilizers were applied twice each year, with 40% in the spring and 60% after pruning.

### Sample collection and preparation

The soil samples (50 cm from the mulberry tree trunk at a depth of 0–20 cm) were collected from the mulberry fields on April 19, 2015 as follows: Da 10 covered (DSC) and uncovered (DSNC), Yunguo 1 covered (YGC) and uncovered (YGNC), Taiwan 72C002 covered (TWC) and uncovered (TWNC), and Dabaie covered (DBC) and uncovered (DBNC). Each sample was a blend of 15 sampling spots that were randomly chosen within each plot. The samples were transported to the lab and were stored at −80 °C for soil microbiological analysis.

### DNA extraction and PCR amplify ITS1

Genomic DNA was directly extracted from the soil using an E.Z.N.A.® Soil DNA kit (Omega Bio-Tec, Inc., USA) according to the manufacturer’s instructions. The quality of the extracted DNA was ensured using 1% agarose gels. The ITS1 regions were PCR amplified from the microbial genomic DNA using the primers ITS1F (5- CTTGGTCATTTAGAGGAAGTAA-3) and ITS2 (5- GCTGCGTTCTTCATCGATGC -3). The reaction mixtures (25 μl) contained 5.0 μl of 5× Q5 reaction buffer (TakaRa, Japan), 5.0 μl of 5× Q5 high enhance, 40 ng of DNA template, 1.0 μl of each primer, 2.0 μl of dNTPs, and 0.25 μl of Q5 polymerase (TakaRa, Japan). The PCR conditions were as follows: 98 °C for 5 min; 27 cycles of 98 °C for 30 s, 56 °C for 30 s, 72 °C for 30 s; and a final elongation at 72 °C for 5 min. The PCR product was excised from a 2% agarose gel and purified using a MinElute PCR Purification Kit (Qiagen, Gmbh, Germany).

Only PCR products without primer dimers and contaminant bands were used for sequencing by synthesis. The cleaned PCR products were sequenced using the paired-end method by Illumina MiSeq with a 7-cycle index read. The data were processed according to the procedure described previously^23^, using the Quantitative Insights into Microbial Ecology (QIIME) pipeline (http://qiime.sourceforge.net)^24^. In brief, the sequences with an average phred score lower than 20, with ambiguous bases, with homopolymer runs exceeding 6 bp, with primer mismatches, or with sequence lengths shorter than 200 bp were removed. Only sequences with an overlap longer than 10 bp and without any mismatch were assembled according to their overlap sequence. The reads that could not be assembled were discarded. Barcode and sequencing primers were trimmed from the assembled sequence^25^.

### Taxonomy classification and statistical analysis

Sequences were clustered and assigned to operational taxonomic units (OTUs) using the QIIME uclust (version 1.8.0) implementation of cd-hit with a threshold of 97% pairwise identity^26^. The longest sequences of the 20 most abundant OTUs were extracted and used as representatives for taxonomic identification by QIIME BLAST searches against the non-redundant Unite sequence database. The OTU abundance of each sample was determined at the genus/species level. The mean length of all effective fungal sequences without the primers was 254 bp. The abundance count at the genus level was log2 transformed and then normalized as follows^27^: from each log transformed measure, the arithmetic mean of all of the transformed values was subtracted, and the difference was divided by the standard deviation of all of the log-transformed values for a given sample. After this procedure, the abundance profiles for all of the samples exhibited a mean of 0 and a standard deviation of 1. The fungal diversity is shown by the number of OTUs. A Venn diagram was generated to compare OTUs between genotypes, and the bacterial community indices applied here included Chao1 and Shannon diversity index. Principal Coordinates Analysis (PCoA) in genus level was performed using Bray-Curtis distances in Mothur 1.29.2. Principal components analysis (PCA) at the genus level was performed using R 2.9.1.

### Data analysis

The results were analysed using SPSS software (version 10.0 for Windows, Chicago, IL, USA). The differences in the abundance of the individual OTUs among genotypes, cover, and genotype by cover interaction were tested by two-way analysis of variance (ANOVA), and the significant differences between the means were determined using the Fisher’s least significant difference test (LSD) with a significance level of p < 0.05. The normal distribution and homogeneity of variance were verified by Bartlett’s and Dunnett’ tests. For all other parameters, data were compared using a one-way ANOVA and T-test. A mean comparison was performed with a significance level of p < 0.05. Most of p-values in T-test were adjusted by FDR using the Benjamini-Hochberg (BH) method by mt.rawp2adjp function in R 2.9.1.

### Accession number of DNA sequence

The raw data has been submitted to a public repository (NCBI) and the accession number was SRP070740, SRX1598154, SRR3186950.

## Additional Information

**How to cite this article**: Yu, C. *et al*. Soil fungal community comparison of different mulberry genotypes and the relationship with mulberry fruit sclerotiniosis. *Sci. Rep.* **6**, 28365; doi: 10.1038/srep28365 (2016).

## Supplementary Material

Supplementary Information

## Figures and Tables

**Table 1:** Comparisons of the incidence of mulberry fruit sclerotiniosis among the susceptible cultivar Da 10 (DS), resistant cultivar Yunguo 1 (YG), Taiwan 72C002 (TW), and Dabaie (DB).

**Mulberry genotypes**	**Incidence of sclerotiniosis (%)**	
	**May 4, 2012**	**April 29, 2015**
DS	25.4 ± 0.04a	36.8 ± 0.03a
YG	0.9 ± 0.02b	4.8 ± 0.01b
TW	0.4 ± 0.01b	6.4 ± 0.03b
DB	2.1 ± 0.04b	3.5 ± 0.01b

Different letters above bars denote statistical significance at p < 0.05, according to LED’s tests.

**Table 2:** Comparisons (T-test) between the resistant cultivar and the susceptible cultivar for phylum abundance.

**Phyla**	**Relative fold change**	p value (*p < 0.05, **p < **0.01)**
	**Resistant cultivar/Susceptible cultivar**	
*Ascomycota*	4.60	0.020
*Basidiomycota*	–1.29	0.040
*Chytridiomycota*	–1.01	0.283
*Glomeromycota*	2.75	0.163
*Rozellomycota*	0.13	0.591
*Zygomycota*	–0.18	0.699

**Table 3:** ANOVA for genus abundance.

**Phyla**	**Genus**	p value (*p < 0.05, **p <0.01)		
		**genotypes**	**cover**	Genotypes*cover
*Ascomycota*	*Mortierella*	0.000**	0.000**	0.000**
	*Humicola*	0.000**	0.002**	0.000**
	*Monographella*	0.000**	0.000**	0.000**
	*Scleromitrula*	0.000**	0.000**	0.001**
	*Scedosporium*	0.000**	0.571	0.000**
	*Staphylotrichum*	0.001**	0.001**	0.000**
	*Tuber*	0.589	0.003**	0.034*
	*Davidiella*	0.369	0.018*	0.060
	*Pleospora*	0.021*	0.147	0.084
	*Spathularia*	0.022*	0.231	0.034*
	*Pseudocercospora*	0.039*	0.047*	0.326
	*Phaeoacremonium*	0.041*	0.025*	0.051
	*Enterographa*	0.044*	0.024*	0.601
	*Microcera*	0.698	0.027*	0.847
	*Penicillium*	0.046*	0.054	0.062
	*Schizothecium*	0.041*	0.071	0.085
	*Chrysosporium*	0.259	0.038*	0.066
	*Sphaerulina*	0.057	0.047*	0.064
	*Fusarium*	0.186	0.042*	0.183
	*Fusicolla*	0.154	0.051	0.048*
*Basidiomycota*	*Guehomyces*	0.349	0.000**	0.000**
	*Conocybe*	0.000**	0.000**	0.000**
	*Inocybe*	0.044*	0.024*	0.601
	*Hyphoderma*	0.034*	0.069	0.658
	*Mrakia*	0.034*	0.021*	0.056
	*Leucopaxillus*	0.044*	0.031*	0.067
	*Ryvardenia*	0.045*	0.021*	0.189
	*Amanita*	0.048*	0.147	0.087
	*Cryptococcus*	0.049*	0.052	0.058
Glomeromycota	*Glomus*	0.044*	0.103	0.055
Chytridiomycota	*Rhizophydium*	0.041*	0.059	0.635

**Table 4:** Genotypes comparisons (T-test) between resistant variety and susceptible variety for genus abundance. The P values were adjusted by FDR using the Benjamini-Hochberg (BH) method.

**Phyla**	**genus**	**Relative fold change**	p value (*p< 0.05, **p<0.01)
		**Resistant variety/Susceptible variety**	
*Ascomycota*	*Humicola*	1.99	0.004**
	*Mortierella*	3.90	0.042*
	*Scleromitrula*	−5.21	0.003**
	*Schizothecium*	4.69	0.045*
	*Sphaerulina*	−1.21	0.049*
	*Lecidella*	4.63	0.047*
*Basidiomycota*	*Cortinarius*	2.78	0.009**
	*Russula*	2.35	0.035*
	*Hymenochaete*	4.40	0.048*
	*Mrakia*	−1.14	0.029*

+: Resistant variety/Susceptible variety

−: Susceptible variety/Resistant variety.

**Table 5:** Comparisons (T-test) between the covered and uncovered treatments for genus abundance.

**Phyla**	**genus**	**Relative fold change**	p value (*p< 0.05, **p < **0.01)**
		**Cover/Uncover**	
*Ascomycota*	*Enterographa*	4.73	0.000**
	*Chrysosporium*	–22.35	0.003**
	*Coniochaeta*	21.13	0.002**
	*Microcera*	–21.45	0.002**
	*Scleromitrula*	–5.51	0.002**
	*Pseudocercospora*	–2.97	0.003**
	*Tuber*	5.62	0.006**
	*Podospora*	–2.84	0.008**
	*Davidiella*	1.78	0.020*
	*Mortierella*	4.22	0.023*
	*Articulospora*	6.61	0.029*
	*Alternaria*	1.49	0.041*
	*Spathularia*	5.95	0.048*
*Chytridiomycota*	*Rhizophydium*	–23.82	0.002**
*Basidiomycota*	*Ryvardenia*	–4.77	0.035*
	*Inocybe*	–5.85	0.038*
	*Conocybe*	–3.13	0.031*
	*Phallus*	3.88	0.005**

The P values were adjusted by FDR using the Benjamini-Hochberg (BH) method.

+: Cover/Uncover

−: Uncover/Cover.
