# Supplementary material for: Soil fungal community comparison of different mulberry genotypes and the relationship with mulberry fruit sclerotiniosis
Source: Sci Rep. 2016 Jun 21;6:28365. doi: 10.1038/srep28365 (PMC4914993; doi:10.1038/srep28365)
Supplement: Supplementary Information [file srep28365-s1.pdf]

Soil fungal community comparison of different mulberry genotypes and the relationship with mulberry fruit sclerotiniosis

Cui Yu, Xingming Hu, Wen Deng, Yong Li, Guangming Han, Chuhua Ye

Industrial Crops Institute of Hubei Academy of Agricultural Sciences, Wuhan 430064, China

Supporting information

Table S1 Raw reads, sequences and OTUs from the susceptible cultivar Da 10 (DS), resistant cultivar Yunguo 1 (YG), Taiwan 72C002 (TW), and Dabaie (DB) with the covered (C) and uncovered (NC) treatments.

Group	Raw read	High quality sequence	OTUs	Sample name	Mulberry genotype	Covered or uncovered	Disease status
DSC1	34,027	30,063	463	Da 10	Susceptible to the sclerotiniosis		
DSC2	39,706	32,183	384			Covered	
DSC3	34,396	30,301	421				
DSNC1	34,806	31,381	451				
DSNC2	38,441	30,866	418			Uncovered	
DSNC3	36,601	29,584	402				
DBC1	37,946	29,491	490	Dabaie	Resistant to the sclerotiniosis		
DBC2	35,149	29,587	646			Covered	
DBC3	30,012	20,654	564				
DBNC1	40,134	30,746	684				
DBNC2	35,491	30,819	461			Uncovered	The disease was occurring in all tested plants
DBNC3	31,230	24,021	521				
TWC1	40,084	32,753	387	Taiwan 72C002	Resistant to the sclerotiniosis		
TWC2	40,253	30,127	429			Covered	
TWC3	36,074	30,174	420				
TWNC1	34,496	28,914	445				
TWNC2	35,106	30,259	577			Uncovered	
TWNC3	38,301	29,564	487				
YGC1	41,338	30,553	410	Yunguo 1	Resistant to the sclerotiniosis		
YGC2	40,870	30,923	550			Covered	
YGC3	31,025	20,054	482				
YGNC1	77,833	70,689	511				
YGNC2	40,353	30,100	445			Uncovered	
YGNC3	40,025	34,048	459				

Table S2 ANOVA for genus abundance

Genus	p value (* p <0.05, ** p <0.01)		
	**genotypes**	**cover**	**Genotypes*cover**
Mortierella	0.000**	0.000**	0.000**
Humicola	0.000**	0.002**	0.000**
Monographella	0.000**	0.000**	0.000**
Scleromitrula	0.000**	0.000**	0.001**
Scedosporium	0.000**	0.571	0.000**
Staphylotrichum	0.001**	0.001**	0.000**
Phaeoacremonium	0.041*	0.025*	0.051
Guehomyces	0.349	0.000**	0.000**
Conocybe	0.000**	0.000**	0.000**
Penicillium	0.046*	0.054	0.062
Schizothecium	0.041*	0.071	0.085
Sphaerulina	0.057	0.047*	0.064
Mrakia	0.034*	0.021*	0.056
Leucopaxillus	0.044*	0.031*	0.067
Fusarium	0.186	0.142*	0.183
Cryptococcus	0.049*	0.052	0.058
Spathularia	0.022*	0.231	0.034*
Hyphoderma	0.034*	0.069	0.658
Microcera	0.698	0.027*	0.847
Glomus	0.044*	0.103	0.055
Pleospora	0.021*	0.147	0.084
Fusicolla	0.154	0.051	0.048*
Davidiella	0.369	0.018*	0.060
Tuber	0.589	0.003**	0.034*
Enterographa	0.044*	0.024*	0.601
Itersonilia	0.027*	0.258	0.321
Ryvardenia	0.045*	0.021*	0.189
Chrysosporium	0.259	0.038*	0.066
Pseudocercospora	0.039*	0.047*	0.326
Rhizophydium	0.041*	0.039*	0.635
Amanita	0.048*	0.147	0.087
Chaetomium	0.071	0.052	0.081
Preussia	0.194	0.169	0.197
Alternaria	0.298	0.259	0.381
Podospora	0.302	0.294	0.310
Oropogon	0.405	0.568	0.529
Clonostachys	0.609	0.841	0.993
Chalara	0.815	0.654	0.708
Lecidella	0.064	0.054	0.068
Hymenochaete	0.257	0.368	0.478

Cortinarius	0.569	0.487	0.681
Russula	0.804	0.478	0.791
Neonectria	0.354	0.158	0.341
Gloeophyllum	0.540	0.159	0.357
Triparticalcar	0.259	0.544	0.301
Inocybe	0.158	0.245	0.298
Helicoma	0.244	0.325	0.308
Pacispora	0.358	0.446	0.612
Devriesia	0.421	0.354	0.741
Powellomyces	0.087	0.091	0.123
Cosmospora	0.088	0.081	0.089
Tricholoma	0.215	0.352	0.055
Geejayessia	0.080	0.259	0.541
Articulospora	0.201	0.327	0.415
Stenella	0.401	0.058	0.213
Ambispora	0.138	0.085	0.357
Lophiostoma	0.301	0.320	0.520
Balansia	0.198	0.421	0.387
Striatosphaeria	0.217	0.357	0.398
Myxocephala	0.214	0.236	0.325
Codinaeopsis	0.091	0.210	0.301
Stephanonectria	0.081	0.136	0.214
Massaria	0.254	0.081	0.365
Trichophaea	0.125	0.065	0.241
Clavulina	0.123	0.061	0.125
Microbotryum	0.087	0.235	0.301
Podosphaera	0.158	0.254	0.269
Nalanthamala	0.214	0.315	0.319
Clavaria	0.148	0257	0.149
Ophiosphaerella	0.249	0.532	0.614
Olpidium	0.214	0.058	0.314
Botrytis	0.189	0.089	0.369
Sonoraphlyctis	0.247	0.358	0.197
Phallus	0.097	0.259	0.129
Ceratobasidium	0.195	0.357	0.698
Viridispora	0.259	0.147	0.352
Pochonia	0.312	0.356	0.246
Petriella	0.235	0.156	0.435
Phialemonium	0.357	0.269	0.268
Auritella	0.328	0.347	0.458
Cladorrhinum	0.821	0.202	0.637
Cylindrocarpon	0.589	0.245	0.269
Talaromyces	0.159	0.214	0.259
Apodospora	0.732	0.598	0.658

Diaporthe	0.547	0.369	0.254
Coniochaeta	0.259	0.317	0.658
Austrolecia	0.654	0.329	0.294
Boothiomyces	0.354	0.267	0.648
Flagelloscypha	0.652	0.294	0.614
Geastrum	0.538	0.351	0.621
Blumeria	0.521	0.354	0.291
Rhodocybe	0.625	0.687	0.954
Deniquelata	0.958	0.647	0.901
Balansia	0.179	0.905	0.934

Table S3 Genotypes comparisons (T-test) between resistant variety and susceptible variety for genus abundance. The P values were adjusted by FDR using the Benjamini-Hochberg (BH) method.

genus	Relative fold change Resistant variety/ Susceptible variety	p value (* p< 0.05, ** p<0.01)
Humicola	1.99	0.004**
Mortierella	3.90	0.042*
Scleromitrula	–5.21	0.003**
Schizothecium	4.69	0.045*
Sphaerulina	–1.21	0.049*
Lecidella	4.63	0.047*
Cortinarius	2.78	0.009**
Russula	2.35	0.035*
Hymenochaete	4.40	0.048*
Mrakia	–1.14	0.029*
Scedosporium	2.22	0.062
Penicillium	–1.84	0.062
Phaeoacremonium	4.92	0.067
Preussia	3.90	0.085
Cryptococcus	2.79	0.143
Coniothyrium	1.54	0.143
Amanita	4.46	0.150
Hyphoderma	4.57	0.154
Ceratobasidium	5.43	0.166
Apodospora	2.29	0.167
Oropogon	2.45	0.202
Glomus	3.00	0.216
Gloeophyllum	3.38	0.234
Talaromyces	–3.04	0.242
Myxocephala	2.53	0.248
Blumeria	3.73	0.250
Geastrum	–2.30	0.253
Tuber	–3.24	0.258
Auritella	3.48	0.258
Podospora	–1.71	0.259
Diaporthe	–3.15	0.260
Cosmospora	1.94	0.263
Neonectria	2.24	0.264
Petriella	2.81	0.280
Pochonia	–2.69	0.282
Tricholoma	–7.65	0.286
Phialemonium	2.71	0.295
Spathularia	3.44	0.302
Coniochaeta	3.30	0.337

Fusicolla	–2.03	0.360
Triparticalcar	–1.13	0.361
Pacispora	–3.54	0.379
Stephanonectria	–2.21	0.388
Clavaria	2.45	0.390
Podosphaera	–3.77	0.397
Devriesia	–4.64	0.416
Fusarium	1.28	0.418
Botrytis	1.66	0.452
Flagelloscypha	4.44	0.480
Rhizophydium	2.23	0.486
Balansia	4.06	0.494
Geejayessia	2.03	0.506
Chaetomium	–0.74	0.532
Cladorrhinum	4.31	0.534
Codinaeopsis	–2.81	0.546
Microcera	–2.45	0.556
Powellomyces	–7.34	0.588
Leucopaxillus	5.02	0.598
Schizophyllum	3.85	0.617
Chrysosporium	4.56	0.648
Conocybe	–1.17	0.658
Rhodocybe	4.08	0.667
Viridispora	1.29	0.670
Deniquelata	–3.77	0.679
Helicoma	–3.29	0.682
Ophiosphaerella	2.92	0.728
Ryvardenia	1.91	0.760
Sonoraphlyctis	–2.38	0.772
Stenella	–1.71	0.780
Davidiella	–0.64	0.791
Articulospora	–1.95	0.806
Alternaria	0.50	0.818
Microbotryum	1.05	0.824
Itersonilia	1.17	0.836
Phallus	–1.49	0.845
Striatosphaeria	–0.78	0.854
Austrolecia	–0.68	0.871
Clavulina	0.36	0.882
Massaria	–1.28	0.889
Enterographa	0.95	0.894
Olpidium	–0.84	0.955
Aspergillus	–0.19	1.000
Pleospora	–0.33	1.000

Lophiostoma	0.55	1.000
Nalanthamala	0.37	1.000
Pseudocercospora	0.22	1.000
Ambispora	0.28	1.000
Cylindrocarpon	0.18	1.000
Clonostachys	–0.15	1.000
Guehomyces	–0.08	1.000
Boothiomyces	–0.15	1.000
Trichophaea	0.04	1.000
Inocybe	0.04	1.000

+: Resistant variety/Susceptible variety

–: Susceptible variety/Resistant variety

Table S4 Comparisons (T-test) between the susceptible cultivar Da 10 (DS), resistant cultivar Yunguo 1 (YG), Taiwan 72C002 (TW), and Dabaie (DB). The P values were adjusted by FDR using the Benjamini-Hochberg (BH) method.

**genus**	**Relative fold change**	**p value**
	**DB / DS**	**(* p <0.05, ** p <0.01)**
Humicola	2.67	0.028*
Enterographa	–1.41	0.041*
Mortierella	3.00	0.045*
Spathularia	–18.19	0.045*
Scleromitrula	–4.77	0.049*
Staphylotrichum	–18.46	0.050*
Hyphoderma	5.94	0.062
Coniothyrium	–3.00	0.062
Geastrum	–3.75	0.088
Botrytis	–1.19	0.091
Leucopaxillus	–0.61	0.098
Clavaria	4.03	0.098
Coniochaeta	4.85	0.136
Triparticalcar	–1.60	0.139
Ophiosphaerella	–19.10	0.144
Oropogon	2.54	0.152
Diaporthe	–3.54	0.156
Blumeria	3.79	0.161
Fusicolla	–2.88	0.168
Striatosphaeria	–2.29	0.180
Mrakia	–1.46	0.192
Talaromyces	–2.55	0.194
Conocybe	–2.00	0.197
Tricholoma	–6.15	0.204
Davidiella	–1.27	0.205
Cryptococcus	3.33	0.218
Cosmospora	2.80	0.244
Tuber	–2.47	0.246
Myxocephala	3.63	0.258
Itersonilia	–0.94	0.260
Podosphaera	–6.87	0.263
Stephanonectria	–2.45	0.278
Apodospora	3.22	0.296
Stenella	–2.92	0.299
Chaetomium	–0.91	0.344
Preussia	1.75	0.350
Devriesia	–4.50	0.355
Sphaerulina	–0.85	0.389
Balansia	5.59	0.410

Petriella	3.01	0.413
Articulospora	–2.40	0.426
Schizophyllum	5.40	0.464
Phaeoacremonium	3.39	0.478
Chrysosporium	–1.19	0.487
Geejayessia	3.58	0.487
Microcera	–2.45	0.487
Phialemonium	1.54	0.487
Rhodocybe	–1.61	0.487
Rhizophydium	1.15	0.487
Pacispora	–2.23	0.539
Powellomyces	–7.23	0.550
Sonoraphlyctis	–5.67	0.554
Fusarium	–1.25	0.565
Hymenochaete	–0.57	0.574
Codinaeopsis	–2.65	0.575
Scedosporium	2.25	0.604
Ryvardenia	3.40	0.605
Alternaria	0.76	0.607
Lecidella	0.88	0.618
Trichophaea	–4.20	0.623
Auritella	3.68	0.652
Cortinarius	–1.19	0.666
Pochonia	–1.47	0.676
Glomus	–1.38	0.727
Microbotryum	–2.16	0.734
Cylindrocarpon	2.47	0.744
Lophiostoma	–2.12	0.746
Pseudocercospora	–1.28	0.748
Viridispora	–1.47	0.758
Deniquelata	–2.37	0.769
Inocybe	–1.98	0.782
Podospora	–0.87	0.786
Aspergillus	0.73	0.809
Helicoma	–1.82	0.826
Russula	1.62	0.834
Phallus	–1.55	0.841
Clonostachys	1.35	0.842
Neonectria	1.79	0.860
Nalanthamala	1.44	0.905
Ambispora	1.36	0.913
Gloeophyllum	–0.74	0.928
Boothiomyces	0.89	0.941
Ceratobasidium	0.24	0.972

Pleospora	0.70	0.995
Penicillium	–0.26	0.997
Flagelloscypha	0.95	1.000
Clavulina	0.24	1.000
Guehomyces	–0.27	1.000
Cladorrhinum	0.27	1.000
Olpidium	0.14	1.000
Amanita	0.18	1.000
Massaria	0.09	1.000
Austrolecia	0.06	1.000

**genus**	**Relative fold change TW / DS**	**p value (* p <0.05, ** p <0.01)**
Aspergillus	–1.52	0.005**
Penicillium	–1.26	0.021*
Scleromitrula	–3.71	0.042*
Hymenochaete	0.92	0.044*
Pleospora	–2.47	0.048*
Humicola	1.72	0.049*
Itersonilia	–1.68	0.013*
Mortierella	1.97	0.062
Sphaerulina	–1.28	0.064
Staphylotrichum	–23.21	0.074
Myxocephala	–1.19	0.072
Viridispora	2.74	0.083
Clonostachys	–1.52	0.090
Pochonia	–5.24	0.114
Scedosporium	1.37	0.140
Diaporthe	–4.88	0.142
Lecidella	–0.69	0.152
Geastrum	–2.94	0.154
Ceratobasidium	6.78	0.155
Clonostachys	5.65	0.164
Podospora	–1.77	0.178
Talaromyces	–2.95	0.184
Stephanonectria	–3.91	0.196
Tuber	–3.08	0.196
Russula	2.13	0.196
Tricholoma	–2.52	0.211
Enterographa	2.44	0.218
Mrakia	–1.38	0.223
Petriella	2.24	0.227
Davidiella	–1.18	0.241
Pacispora	–5.58	0.253
Podosphaera	–2.48	0.269

Glomus	–2.34	0.282
Cosmospora	–1.44	0.286
Cortinarius	2.05	0.298
Apodospora	4.59	0.308
Oropogon	3.37	0.330
Spathularia	4.66	0.336
Cryptococcus	3.37	0.341
Schizophyllum	–0.82	0.349
Preussia	4.39	0.353
Devriesia	–4.45	0.365
Striatosphaeria	1.18	0.389
Microbotryum	–4.85	0.406
Flagelloscypha	5.96	0.432
Hyphoderma	3.32	0.440
Helicoma	–6.76	0.444
Cladorrhinum	5.85	0.450
Rhizophydium	2.79	0.455
Lophiostoma	–4.44	0.467
Chrysosporium	–1.19	0.488
Trichophaea	–2.74	0.488
Coniochaeta	–1.19	0.488
Phaeoacremonium	–1.61	0.488
Microcera	–2.45	0.488
Phialemonium	2.04	0.488
Rhodocybe	–1.61	0.488
Powellomyces	–2.32	0.488
Amanita	–1.19	0.539
Clavaria	–1.19	0.539
Leucopaxillus	6.58	0.542
Gloeophyllum	–1.46	0.545
Deniquelata	–5.11	0.586
Blumeria	4.64	0.602
Ophiosphaerella	4.51	0.613
Massaria	–2.60	0.688
Neonectria	–2.93	0.695
Fusicolla	–1.14	0.720
Codinaeopsis	–1.91	0.725
Botrytis	–0.59	0.769
Inocybe	1.47	0.846
Alternaria	0.29	0.864
Clavulina	0.29	0.883
Pseudocercospora	–0.32	0.924
Olpidium	–0.89	0.946
Balansia	0.74	0.947

Cylindrocarpon	0.96	0.950
Ryvardenia	–0.50	0.952
Guehomyces	0.42	0.960
Sonoraphlyctis	–0.93	0.966
Austrolecia	–0.52	0.978
Phallus	–0.65	1.020
Articulospora	–0.36	1.000
Chaetomium	–0.14	1.000
Boothiomyces	–0.25	1.000
Ambispora	–0.20	1.000
Fusarium	0.08	1.000
Auritella	0.18	1.000
Stenella	–0.13	1.000
Nalanthamala	0.12	1.000
Conocybe	–0.07	1.000
Triparticalcar	0.02	1.000
Geejayessia	0.01	1.000

**genus**	**Relative fold change YG / DS**	**p value (* p<0.05, ** p<0.01)**
Humicola	4.00	0.000**
Scleromitrula	–5.46	0.012*
Coniothyrium	2.66	0.031*
Mortierella	5.96	0.025*
Preussia	4.35	0.025*
Penicillium	–1.22	0.027*
Lecidella	6.17	0.031*
Pseudocercospora	–1.97	0.037*
Staphylotrichum	–3.43	0.045*
Amanita	6.02	0.039*
Glomus	4.55	0.048*
Triparticalcar	–2.46	0.051
Ryvardenia	–5.43	0.062
Podospora	–3.69	0.067
Gloeophyllum	4.92	0.071
Microbotryum	2.57	0.071
Russula	2.98	0.078
Sphaerulina	–1.60	0.079
Chaetomium	–1.50	0.083
Clonostachys	–1.46	0.085
Fusarium	2.54	0.096
Neonectria	3.40	0.109
Conocybe	–2.94	0.116
Cryptococcus	–1.45	0.118
Tuber	–5.73	0.120

Oropogon	–1.77	0.126
Ceratobasidium	4.23	0.127
Talaromyces	–3.91	0.132
Pochonia	–3.66	0.139
Ophiosphaerella	–1.10	0.154
Austrolecia	–2.22	0.162
Scedosporium	2.73	0.168
Fusicolla	–2.80	0.174
Nalanthamala	–3.94	0.180
Tricholoma	–2.52	0.194
Myxocephala	2.30	0.197
Botrytis	3.07	0.202
Trichophaea	1.59	0.210
Apodospora	0.52	0.217
Aspergillus	–0.70	0.218
Pacispora	–5.44	0.220
Cortinarius	1.16	0.246
Cosmospora	2.18	0.247
Diaporthe	–2.21	0.276
Codinaeopsis	–2.48	0.277
Stenella	–2.92	0.278
Schizophyllum	–0.86	0.287
Hymenochaete	–1.25	0.311
Devriesia	–5.00	0.322
Olpidium	–4.66	0.326
Massaria	–2.80	0.371
Lophiostoma	–1.82	0.383
Phialemonium	2.92	0.392
Articulospora	–2.40	0.403
Inocybe	–4.03	0.427
Helicoma	–6.20	0.451
Auritella	4.28	0.466
Deniquelata	–2.30	0.486
Balansia	–1.19	0.486
Geejayessia	1.87	0.486
Microcera	–2.45	0.486
Petriella	1.87	0.486
Phaeoacremonium	–1.61	0.486
Hyphoderma	–1.61	0.486
Rhizophydium	1.10	0.486
Boothiomyces	–2.82	0.486
Enterographa	–2.22	0.534
Clavaria	–1.19	0.534
Geastrum	–1.32	0.554

Phallus	–3.51	0.563
Powellomyces	–6.39	0.569
Chrysosporium	6.14	0.576
Sonoraphlyctis	–5.07	0.586
Rhodocybe	5.66	0.590
Podosphaera	–2.25	0.606
Itersonilia	2.57	0.664
Alternaria	1.29	0.683
Spathularia	2.85	0.690
Leucopaxillus	–0.24	0.707
Stephanonectria	–1.33	0.707
Viridispora	–1.75	0.744
Ambispora	–2.23	0.762
Mrakia	–0.72	0.806
Striatosphaeria	0.63	0.860
Cladorrhinum	–0.88	0.870
Pleospora	–0.78	0.877
Guehomyces	–0.59	0.946
Clavulina	0.52	0.998
Cylindrocarpon	0.36	1.000
Coniochaeta	–0.32	1.000
Flagelloscypha	–0.32	1.000
Davidiella	0.09	1.000
Blumeria	–0.10	1.000

+: DB/DS, TW/DS, YG/DS

–: DS/DB, DS/TW, DS/YG

Table S5 comparisons (T-test) between cover and uncover treatments for genus abundance. The P values were adjusted by FDR using the Benjamini-Hochberg (BH) method.

Genus	DS<br>Cover / No cover<br>(* p< 0.05, ** p<0.01)	DB<br>Cover / No cover<br>(* p< 0.05, ** p<0.01)
Humicola	0.000**	0.217
Mrakia	0.421	0.136
Chaetomium	0.987	0.969
Guehomyces	0.120	0.132
Conocybe	0.000**	0.992
Penicillium	0.023*	0.051
Scleromitrula	0.004**	0.024*
Fusarium	0.059	0.044*
Alternaria	0.321	0.231
Cryptococcus	0.000**	0.036*
Oropogon	0.892	0.006**
Lecidella	0.302	0.151
Clonostachys	0.002**	0.218
Podospora	0.000**	0.099
Schizophyllum	0.423	0.187
Leucopaxillus	0.015*	0.068
Stephanonectria	0.050*	0.019**
Apodospora	0.306	0.180
Tuber	0.000**	0.168
Botrytis	0.305	0.612
Mortierella	0.034*	0.100
Fusicolla	0.230	0.330
Talaromyces	0.256	0.056
Aspergillus	0.705	0.908
Hymenochaete	0.007**	0.062
Scedosporium	0.261	0.292
Sphaerulina	0.123	0.194
Gloeophyllum	0.231	0.193
Clavulina	0.398	0.004**
Cladorrhinum	0.531	0.114
Viridispora	0.059	0.421
Olpidium	0.043*	0.035*
Austrolecia	0.008**	0.159
Ceratobasidium	0.628	0.534
Davidiella	0.058	0.003**
Cylindrocarpon	0.231	0.192
Pseudocercospora	0.009**	0.002**
Coniothyrium	0.005**	0.092
Ryvardenia	0.214	0.194

Microbotryum	0.226	0.021**
Triparticalcar	0.596	0.103
Preussia	0.217	0.824
Itersonilia	0.156	0.316
Rhizophydium	–	0.190
Sonoraphlyctis	0.192	0.958
Helicoma	0.184	0.185
Lophiostoma	0.168	0.163
Cortinarius	0.754	1.114
Striatosphaeria	0.024*	0.230
Pleospora	0.956	0.284
Phialemonium	–	0.181
Phallus	0.124	0.068
Glomus	0.596	0.190
Powellomyces	0.190	0.781
Ophiosphaerella	0.462	–
Boothiomyces	0.185	0.389
Devriesia	0.218	0.425
Nalanthamala	0.045*	0.196
Stenella	0.278	–
Russula	0.114	0.214
Tricholoma	0.098	0.190
Neonectria	0.190	0.190
Inocybe	0.212	0.190
Chrysosporium	0.190	–
Trichophaea	0.190	0.697
Ambispora	0.212	0.203
Spathularia	0.893	–
Articulospora	0.175	–
Balansia	0.190	0.202
Flagelloscypha	0.190	0.190
Cosmospora	0.318	0.288
Phaeoacremonium	0.190	0.139
Schizothecium	0.190	0.004**
Petriella	–	0.137
Geejayessia	–	0.190
Enterographa	0.190	1.190
Massaria	0.215	0.061
Amanita	0.190	0.190
Codinaeopsis	0.216	0.218
Hyphoderma	0.190	0.734
Pochonia	0.021*	0.190
Podosphaera	0.320	0.190
Geastrum	0.000**	0.191

Myxocephala	0.824	0.212
Pacispora	0.046*	0.220
Blumeria	0.190	0.107
Coniochaeta	0.190	0.083
Microcera	0.190	–
Auritella	0.190	0.190
Deniquelata	0.190	0.079
Diaporthe	0.000**	0.749
Rhodocybe	0.190	–
Clavaria	0.190	0.866

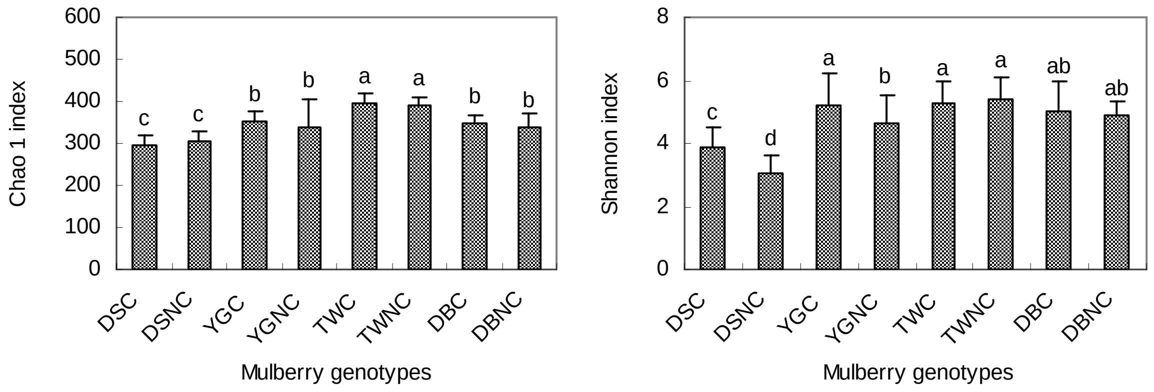

a **b**

Fig. S1 (a) The fungal abundance of Chao1 index of the susceptible cultivar Da 10 (DS), resistant cultivar Yunguo 1 (YG), Taiwan 72C002 (TW), and Dabaie (DB) with the covered (C) and uncovered (NC) treatments. (b) Shannon index of different mulberry genotypes with cover and uncover treatments. Error bars indicate standard deviation (SD) (n=3). Different letters above bars denote statistical significance at $p < 0.05$, according to LED's tests.

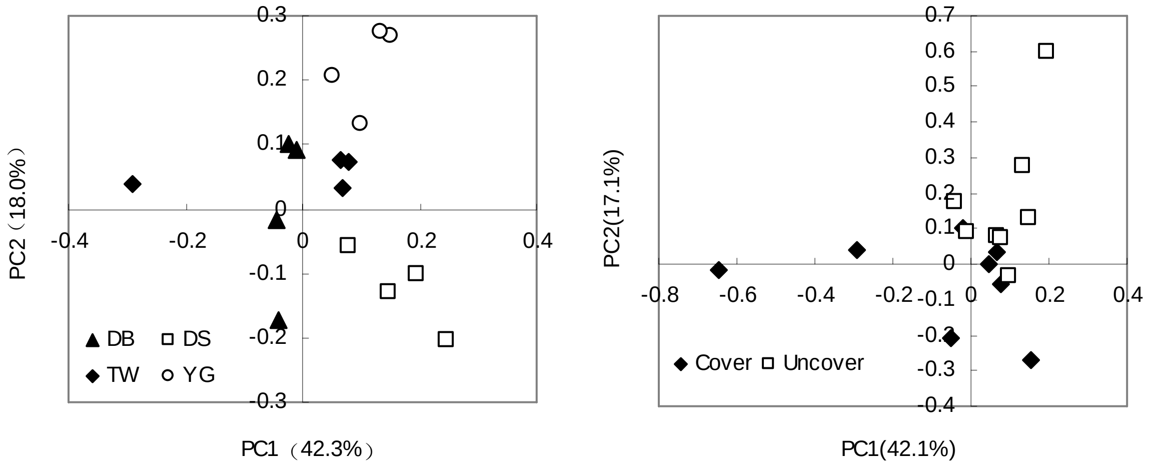

a **b**

Fig. S2 (a) The principal components analysis (PCA) of 16 samples from the different genotypes using 31 soil fungal genera, which were affected by genotypes. (b) PCA of 16 samples from the covered and uncovered soil treatments using 20 fungi genera, which were affected by genotypes.

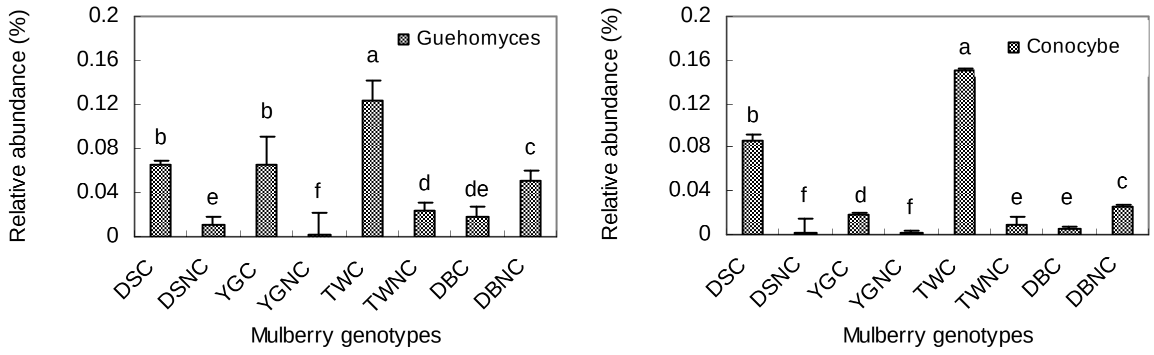

a **b**

Fig. S3 Relative abundances of *Guehomyces* (a) and *Conocybe* (b) of the susceptible cultivar Da 10 (DS), resistant cultivar Yunguo 1 (YG), Taiwan 72C002 (TW), and Dabaie (DB) with the covered (C) and uncovered (NC) treatments. Error bars indicate standard deviation (SD) (n=3). Different letters above bars denote statistical significance at $p < 0.05$, according to LED's tests.
